# Investigation the effect of different ionic liquids based-aryl imidazole on the onset precipitation of asphaltene

**DOI:** 10.1038/s41598-023-31066-0

**Published:** 2023-03-11

**Authors:** Raghda A. El-Nagar, Maher I. Nessim, Dina A. Ismail, Manal G. Mohamed, Alaa Ghanem

**Affiliations:** 1 Petroleum Testing Lab, Analysis and Evaluation Department, Egyptian Petroleum Research Institute, Nasr City, Cairo, 11727 Egypt; 2 Surface Active Agent Lab, Petrochemical Department, Egyptian Petroleum Research Institute, Nasr City, Cairo, 11727 Egypt; 3 Polymer Lab, Petrochemical Department, Egyptian Petroleum Research Institute, Nasr City, Cairo, 11727 Egypt; 4 PVT Lab, Production Department, Egyptian Petroleum Research Institute, Nasr City, Cairo, 11727 Egypt

**Keywords:** Ionic liquids, Fossil fuels, Crude oil, Petrol

## Abstract

Precipitation and deposition of asphaltene are considered as catastrophic issues facing the petroleum industry. Asphaltene deposition mainly occurs at variety places such as formation pore spaces, pumps, pipelines, wellbore, wellhead, tubing, surface facilities and safety valves causing operational problems, production deficiencies and enormous economic losses. This work aims to study the effect of series of synthesized aryl ionic liquids (ILs) containing different alkyl chains, named as R_8_-IL, R_10_-IL, R_12_-IL, and R_14_-IL, on the onset precipitation point of asphaltene in crude oil. R_8_-IL, R_10_-IL, R_12_-IL, and R_14_-IL were synthesized with high yields (the yield varied between 82 and 88%) and characterized via different tools of analysis (FTIR, ^1^H NMR, and Elemental Analysis). Their Thermal Gravimetric Analysis (TGA) was investigated and showed a reasonable degree of stability. It was found that R_8_-IL (short alkyl chain) has the highest stability, while R_14_-IL (long alkyl chain) is the lowest one. Quantum chemical calculations were conducted to study the reactivity and geometry of their electronic structures. Moreover, surface and interfacial tension of them were studied. It was found that the efficiency of the surface active parameters increased by increasing the length of the alkyl chain. The ILs were evaluated to delay the onset precipitation point of asphaltene using to different methods; the kinematic viscosity and the refractive index. Results from the two methods showed delaying of onset precipitation after the addition of the prepared ILs. The asphaltene aggregates was dispersed due to the π–π* interactions and hydrogen bonds formation with the ILs.

## Introduction

Crude oil still plays a significant role in the energy domain, although researchers are seeking different sources of energy because of high demand^1,2^. The use of primary and secondary oil recovery techniques leaves more than 30% of the oil unrecovered inside the pores of the reservoirs. Asphaltene is the heaviest and most aromatic component of crude oil; it is critical to overall aspects in upstream or downstream operations because of its nature to coordinate and form clusters^3^. The viscosity of crude oil is strongly affected by asphaltene; consequently, all areas of resource exploitation are affected, including flow assurance, low distillate, and emulsion stability, resulting in wettability and phase separation problems. According to its solubility, asphaltene is insoluble in short alkane chains and completely soluble in aromatics, e.g., benzene, toluene, and xylene (BTX)^4^. Different asphaltene inhibition treatments have been improved to enhance the properties of crude oil: carbon rejection technologies; solvent deasphalting (SDA); mild cracking solvent deasphalting (MCSD); and the aquathermolysis method. The aquathermolysis method has been reported as the most effective technique for viscosity reduction in heavy crude oil, increasing saturates and aromatics while decreasing resin and asphaltene. Additionally, it requires a severe amount of energy and causes environmental hazards^5,6^. As a matter of fact, resin in crude oil serves as asphaltene inhibitor because of its functional groups and alkyl chains have the ability to link between the asphaltene and nonpolar medium^7,8^. Many synthesized chemicals that have similar structure to resins can enhance the asphaltene stabilization in the system. Most of the reported chemicals that have been used as potential asphaltene dispersants include oxazolidines^6^, n-aryl amino alcohol^9^, benzoic acid, phthalic acid, and salicylic acid^10^. All these chemicals are toxic compounds that may cause many environmental hazards. From this point, ionic liquids (ILs) as a new eco-friendly class of chemicals were suggested by researchers^11,12^. ILs have attracted significant interest in a wide variety of industrial applications because of their distinctive characteristics and great compatibility with environmental issues^13^. Negligible vapor pressure, recyclability, high thermal stability, non-corrosive, high surface activity, and slightly lower toxicity are all appropriate properties for ILs to be considered as environmentally preferable and better sustainable the conventional surfactant^14–18^. The ILs properties owning to poor coordination combination between cations and anions which make possible alterations in the chemical structures subsequently, they can perform better in different applications^19,20^; enhanced oil recovery^21–23^, scale removal, catalysis, capturing of CO_2_^24^, solvent extraction^25^, electrochemistry, natural gas purification^26^, desulphurization, crude oil dissolution and IFT reduction^27,28^. ILs were reported by Liu et al.^29^ for the first time in asphaltene dissolution, and it was noticed that the most effective ILs contained conjugated aromatic cations and anions with strong hydrogen bond acceptors. Meanwhile, there was another work reported by Boukherissa et al. on the usage of boronic ILs (1-propyl boronic acid-3- alkylimidazolium bromide) in asphaltene dispersion^30^. They predicted that the boronic acid moiety would reduce asphaltene aggregation and improve interactions between asphaltenes and ionic liquids. Also, acidic IL (3-(2-carboxybenzoyl)-1-methyl-1H-imida zol-3-ium chloride) was reported to prevent flocculation of asphaltenes^31^. Ghanem et.al. reported the effect of alkylated imidazoleum sulfonate ILs as effective asphaltene dispersants^7^. Protic ILs can cause the dissolution of asphaltenes via cation interactions and charge transfer to form complexes with asphaltene molecules. They all concluded that the electrostatic interaction and hydrogen bond formation promoted avoiding asphaltene accumulation^32^.

This work aims to synthesize and study the effect of some aryl imidazolium ionic liquids containing different alkyl chains, named R_8_-IL, R_10_-IL, R_12_-IL, and R_14_-IL, on the onset precipitation point of asphaltene in crude oil. They were synthesized with high yields, (the yield varied between 82 and 88%), and characterized via different tools of analysis (FTIR, 1H NMR, and Elemental Analysis). In addition, surface tension study was conducted to evaluate the activity of the prepared ionic liquids. The crude oil was characterized according to standard methods. The prepared ionic liquids were evaluated as asphaltene dispersant using the kinematic viscosity and the refractive index technique.

## Experimental work

### Materials

1*H*-Imidazole(≥ 99%), 1-Bromo Octane (≥ 99%), 1-bomodecane(≥ 98%), 1-bromododecane (≥ 99%), 1-bomotetradecane (≥ 99%), 1-(chloromethyl)-4-methylbenzenepotassium hydroxide (≥ 98%), heptanes (≥ 97%), benzene (≥ 98%), n-hexane (≥ 99%), aluminum oxide (neutral) and chloroform (≥ 98%) were supplied by Biochem. All the used chemicals and solvents were of analytical grade and were used with no further purifuication. The used heavy crude oil in this study was received from the Egyptian Petroleum Company. Different physical characteristics and the SARA analysis are tabulated in Table 1. Most of the physico-chemical properties and SARA test of the crude oil were conducted depending on standards of the American Society for Testing and Material (ASTM). Results indicate that the crude oil has a low API degree, a high value of kinematic viscosity, in addition to a high content of asphaltene and a low content of saturates.

**Table 1:** Physico-chemical properties of crude oil^11^.

Experiment	Method	Result
**Density (at 15.6 °C)**	ASTM D5002	0.9499
**Specific gravity**		0.9508
**API **^**o**^		16.3
**Asphaltene content, wt%**	IP-143	13.68
**Kinematic Viscosity @40 °C, cSt**	ASTM D-445	1699.81
**Chemical composition, wt.%**		
**Maltene**	ASTM D-2007	86.32
Saturate		36.91
Aromatic		32.48
Resin		16.93
**Asphaltene**		13.68

### Methodology

**Figure 1:**
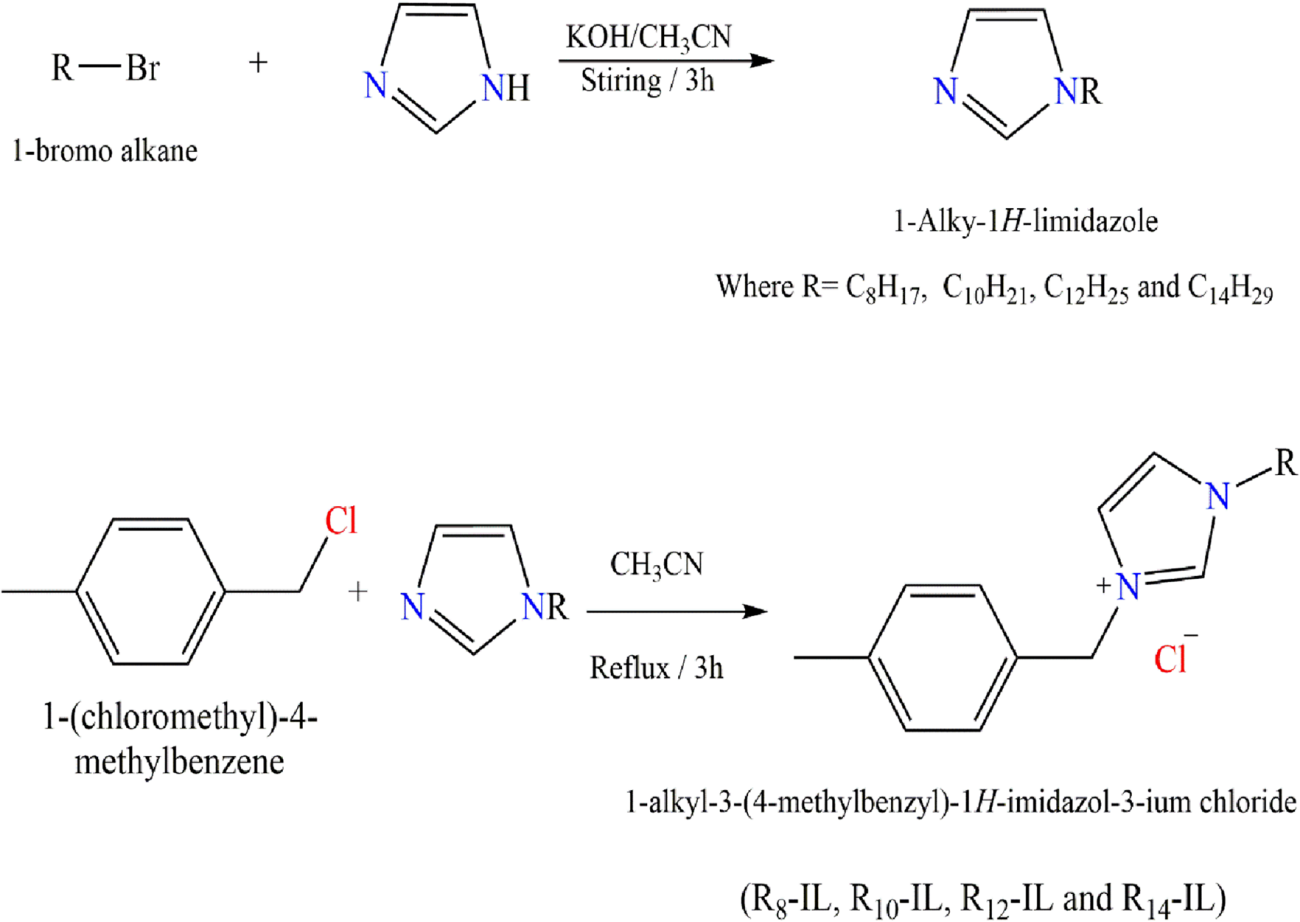
Synthesis of R_8_-IL, R_10_-IL, R_12_-IL, and R_14_-IL.

Synthesis of R_8_-IL, R_10_-IL, R_12_-IL, and R_14_-IL was conducted where, a series of four alkyl imidazole were obtained by adding a known portion of 1H-imidazole (0.1 mol) and potassium hydroxide (KOH) in acetonitrile (50 ml) with continuous stirring. 1-bromo octane, 1-bomo decane,1-bromo dodecane and 1-bomo tetradecane (0.1 mol) was added dropwise to the previous mixture for 3 h untill the formation of a white precipitate from KCl. After filtration process, the filtrate was concentrated under vaccum and different alkyl imidazole ionic liquids were obtained^11^. The four 1-alkyl imidazole derivatives were refluxed with 1-(chloromethyl)-4-methylbenzene in acetonitrile for six hours to yield 1-alkyl-3-(4-methylbenzyl)-*H*-imidazol-3-ium chloride derivetives^26^, as shown in Fig. 1. The TLC technique was used to confirm the purity of the prepared ionic liquids. These compounds showed good solubility in varities of polar solvents^33^.

### Characterization of the prepared ILs

The prepared ILs were characterized using different tools of analysis. Elemental analysis was determined using elementar analyzer, in which an excess of oxygen is used to burn the sample. FTIR spectra of the prepared ILs were analysed in transmittance mode using KBr pellets in the range of 4000–400 cm^-1^ by the Nicolet IS-10 Spectrometer-ThermoFisher, USA. The FTIR spectrometer was equipped with a standard fast recovery deuterated triglycine sulfate detector (DTGS). ^1^H-NMR spectra of the prepared ionic liquids were investigated using BRUKER NMR Spectroscopy, (Germany) using DMSO as a solvent at frequency equal to 400 MHz. Thermal Analyzer SDT Q500 V20.10 with heating rate of 10 ºC/min was used to determin the thermal gravimetric analysis of the prepared ionic liquid. Surface and Interfacial tension of the ionic liquids were investigated at 298 K by Du-Nouy tensiometer depending on a platinum ring. The apparatus was firstly calibrated using deionized water, which recorded about 72 ± 0.5 m/Nm^34^. After that solutions containing a series concenterations of ILs (0.001–0.00001 mol/L) were prepared and measured and different parameters were calculated.

### Asphaltene isolation from crude oil

In this work the asphaltene was isolated from heavy crude oil come from Egyptian oilfield exactly as reported in IP-143 as a standared test method. Aknown portion of crude oil (10 g) was added to 300 ml n-heptane and refluxed for an hour. The refluxed solution was left in dark place until cooling. After that the solution was poured on an ashless filter paper to separate the filterate that contains asphaltene, inorganic and waxy substances. The filter paper was folded many times and entered to a soxhelet to be cleaned with hot n-heptane from any impurities or waxy components. After that the asphaltene was isolated from any inorganic substances via disolvation in hot toluene^35^. At the end, the toluene was evaporated and the asphatene was weighted to determine the asphaltene content according to the following equation.

1 $$A = \frac{M}{G}*100$$

where M is the asphaltene mass and G is the crude oil mass.

### Asphaltene onset precipitation

The evaluation of the onset precipitation point of asphaltene is often conducted by the viscometric method, in which the crude oil is treated with a series of successive volumes of asphaltene precipitant like n-heptane^36^. For each run, series of samples were prepared to cover the precipitant concentration range of 0–100 vol percent from the oriinal crude oil, in the absence and presense of the prepared ionic liquids. The concentration range of the added dispersants was of 500–2000 ppm. After that the mixtures were settled for a period of time till equilibrium. The kinematic viscosities of the prepared mixtures are determined using cannon fensky viscometer at 40 °C. A relation between the the kinematic viscosity and the concentration of the precipant are being drawn to detect the onset precipitation point of asphaltene. The previous steps are repeated after the addition of the dispersants to be evaluated^7^. The refractive index method can be used to confirm the obtained results from the viscometric method. This method uses the asphaltene itself to design a model oil with n-heptane/toluene mixture (Heptol mixture)^37^.

## Results and discussion

### Characterization of the ILs

Table 2 contains the molecular structures of the prepared ionic liquids. The molecular weight of R_8_-IL, R_10_-IL, R_12_-IL, and R_14_-IL is 320.90, 348.95, 377.01, and 405.06, respectively, while the yield varied between 82 and 88%. The reaction yield was calculated according to the following equation:

**Table 2:** The molecular structure of the ILs.

ILs	Molecular structure	Yield
R_8_-IL		83.1
R_10_-IL		82.4
R_12_-IL		88.0
R_14_-IL		85.6

$${\text{Yield}}\;\% = \left( {{\text{Actual}}\;{\text{ yield}}/{\text{Theoretical}}\;{\text{yield}}} \right) \times {1}00.$$

### Elemental analysis

The elements content of the prepared ILs provides information about the role of the organic molecules. The molecular formula can be confirmed by comparing the theoretical data with the experimental ones.The data in Table 3 showed that the calculated C, H, N, O, and S values are compatible with the recorded values.

**Table 3:** Elemental analysis of R_8_-IL, R_10_-IL, R_12_-IL, and R_14_-IL.

Elements								
ILs	C%		H%		N%		Cl %	
	Calculated	Observed	Calculated	Observed	Calculated	Observed	Calculated	Observed
R_8_-IL	71.11	71.08	9.11	9.08	8.73	8.78	11.05	11.06
R_10_-IL	72.28	72.35	9.53	9.56	8.03	8.00	10.16	10.09
R_12_-IL	73.27	73.25	9.89	9.92	7.43	7.39	9.40	9.44
R_14_-IL	74.13	74.17	10.20	10.14	6.92	6.98	8.75	8.71

### FT-IR spectra of the prepared ILs

**Figure 2:**
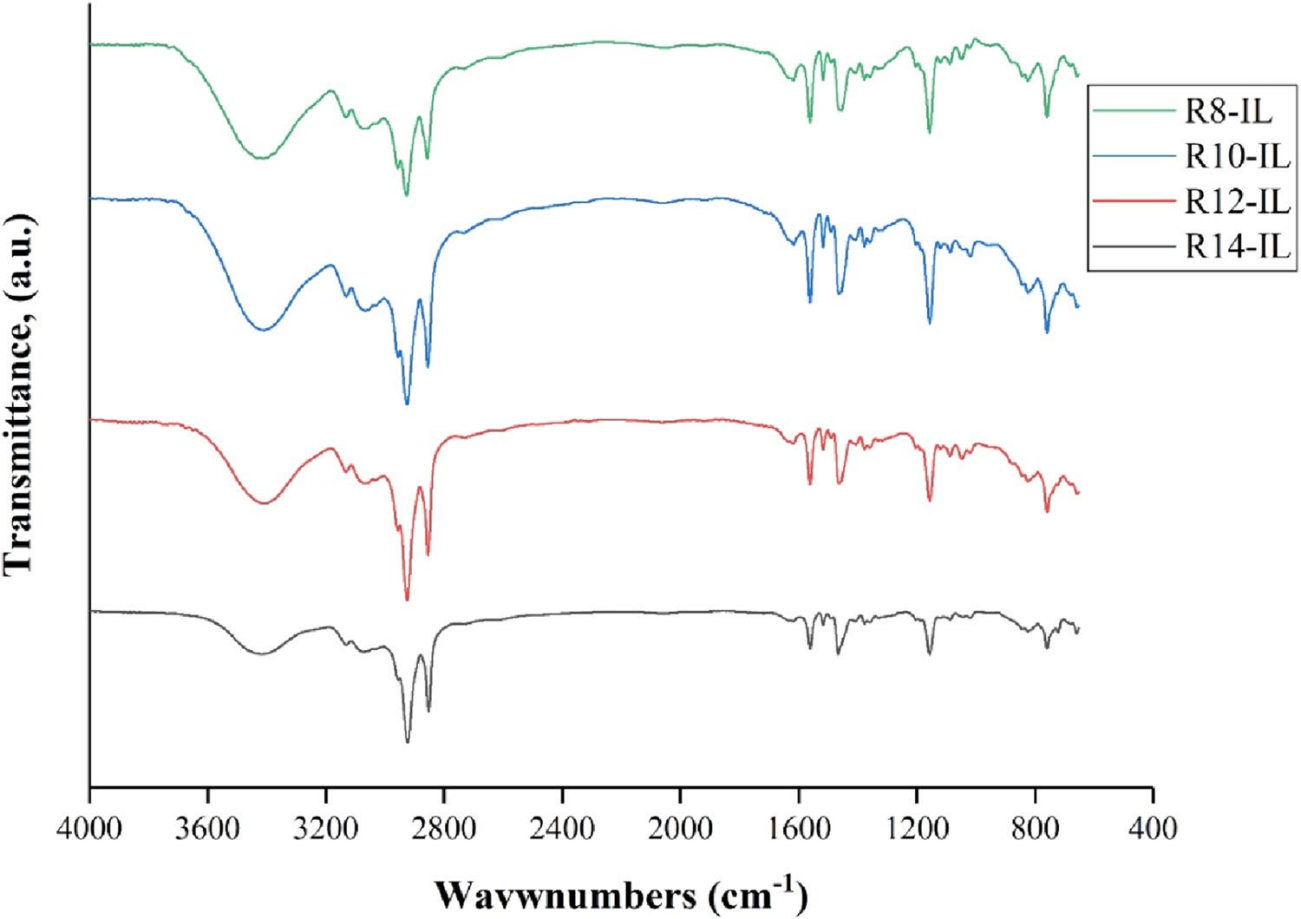
FTIR spectra for the prepared ionic liquids.

The FT-IR spectra of R_8_-IL, R_10_-IL, R_12_-IL, and R_14_-IL are shown in Figure 2, and the values of the characteristic peaks are reported in Table 4. The asymmetric stretching vibrational mode belongs to the hydrogen bonded H_2_O molecules was recorded as a broad band around 3408–3420 cm^−1^^38^. The peaks that appeared between 3131 and 3073 cm^−1^ are attributed to aromatic C–H, and related to the vibrational motion inside the imidazole ring. Aliphatic C–H stretching peaks appeared in the range of 2956 and 2852 cm^−1^, where the peaks that were observed around 2956 and 2925 cm^−1^ are attributed to the asymetric stretching of the terminal methyl group and the methylene unit in the alkyl chain of the prepared ionic liquids, respectively. In addition, the symmetric stretching peaks of CH_3_ and CH_2_ appeared around 2872 and 2852 cm^−1^, respectively. The symmetric and asymmetric vibrational modes are usually assigned between 2954 and 2870 cm^−1^ which become more intense peaks by increasing the alkyl chain length by more than six CH_2_ groups^39^. The peaks that appeared between 1561 and 1517 cm^−1^ are attributed to C–C vibration inside the imidazole ring. The appeared peaks between 1466 and 1456 cm^−1^ are belonged to C=C aromatic. Stretching vibration modes that resorted to C–N in the imidazole ring appeared at 1377 cm^−1^. Another stretching vibrational peaks appeared at 1157–1156 cm^−1^ are attributed to C−H in plane.

**Table 4:** FT-IR data of the prepared ILs.

ILs	CH–H2O	C − H aromatic	C − H aliphatic	C–C aromatic	C = C aromatic	C − N	C − H in plane	C − H out of plane
R_8_-IL	3411	3131	2956–2856	1561–1517	1456	1377	1157	759
R_10_-IL	3408	3130	2925–2854	1561–1517	1463	1377	1156	759
R_12_-IL	3409	3131	2925–2854	1561–1517	1463	1377	1156	759
R_14_-IL	3420	3073	2923–2852	1561–1517	1466	1377	1157	759

### ^1^H-NMR of the prepared ILs

The proton NMR spectra for R_8_-IL, R_10_-IL, R_12_-IL, and R_14_-IL were studied and showed approximately nearby chemical shifts, as shown in Figures S1, S2, S3, and S4 in the supplementary materials. The adjacent chemical shifts of each ionic liquid can be concluded as follows:

#### For R_8_-IL

δ 9.29 (1H, s), 7.81 (2H, d), 7.79 (2H, d), 7.69 (2H, s), 5.42 (2H, s), 4.16 (2H, t), 3.37 (3H, s), 2.51 (2H, t), 1.79 (8H, m), 1.24 (2H, s), 0.86 (3H, t).

#### For R_10_-IL

δ 9.19 (1H, s), 7.78 (2H, d), 7.70 (2H, d), 7.47 (2H,s), 5.43 (2H, s), 4.17 (2H, t), 3.38 (3H, s), 2.51 (2H, t), 1.79 (12H, m), 1.25 (2H, s), 0.86 (3H, t).

#### For R_12_-IL

δ 9.20 (1H, s), 7.80 (2H, d), 7.69 (2H, d), 7.46 (2H, s), 5.43 (2H, s), 4.16 (2H, t), 3.39 (3H, s), 2.51 (2H, t), 1.79 (16H, m), 1.24 (2H, s), 0.86 (3H, t).

#### For R_14_-IL

δ 9.50 (1H, s), 7.85 (2H, d), 7.67 (2H, d), 7.34 (2H, s), 5.41 (2H, s), 4.17 (2H, t), 3.41 (3H, s), 2.28 (2H, t), 1.76 (20H, m), 1.24 (2H, s), 0.88 (3H, t).

From the above data, the distinct chemical shifts at δ 9.2 (1H, s) displayed the characteristic peaks of the imidazolium protons and δ 5.4 (2H, s) are attributed to the benzylic protons of alkyl imidazole, demonstrating the effective incorporation of imidazolium group.

### Thermal gravimetric analysis (TGA)

**Figure 3:**
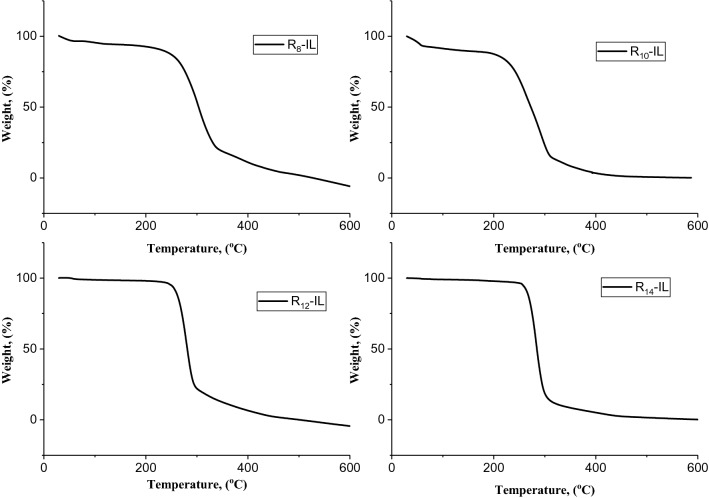
TGA thermograms of the synthesized ionic liquids.

Thermal gravimetric analysis thermograms of R_8_-IL, R_10_-IL, R_12_-IL, and R_14_-IL are shown in Fig. 3, and their main measuring values are reported in Table 5. It is obvious that the prepared compounds are thermally stable and R_8_-IL is the most thermaly stable compound, followed by R_10_-IL, R_12_-IL, and R_14_-IL. This may refered to the variation in the length of the alkyl chains that were connected to imidazolium rings of the synthetic compounds^40^. In breif, it is obvious that the maximum thermal stability was associated with the shortest alkyl chain connected to the imidazolium ring^40,41^.

**Table 5:** Characteristic TGA results of the synthesized ionic liquids.

Compound	R_8_-IL	R_10_-IL	R_12_-IL	R_14_-IL
First degradation, ^o^C	320	310	290	260
Final degradation, ^o^C	500	450	475	480

### Quantum chemical parameters

**Figure 4:**
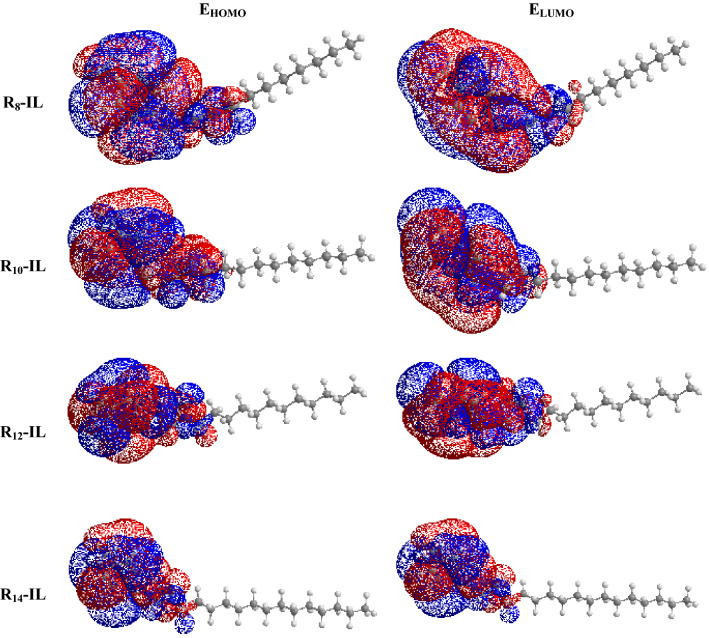
Molecular orbital structures of the prepared compounds.

**Figure 5:**
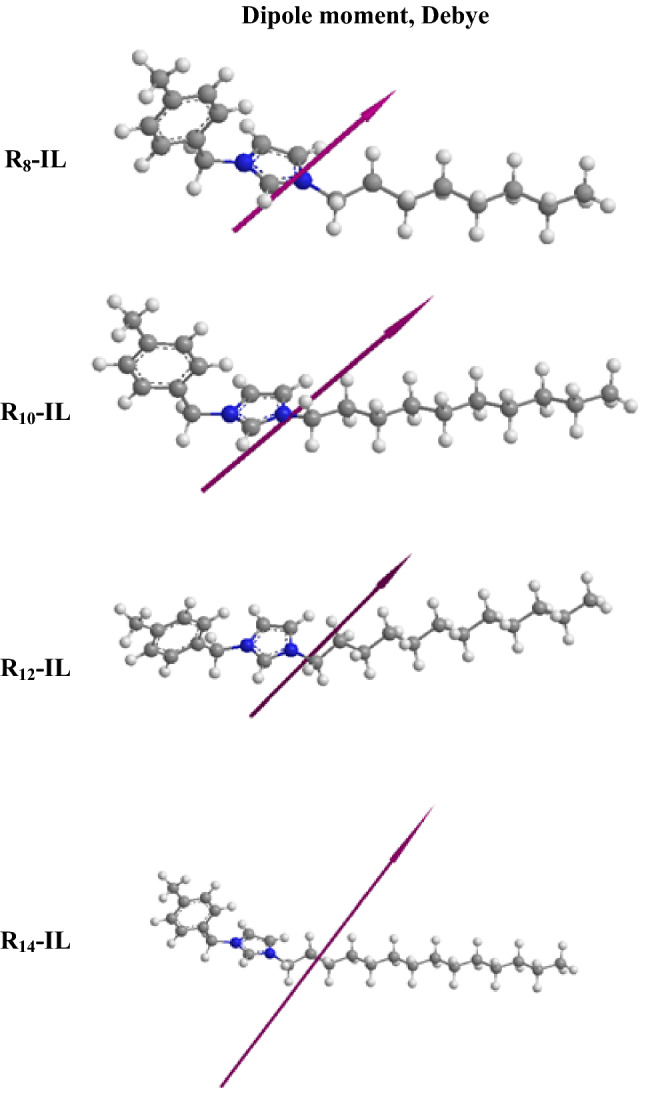
Dipole moments of the prepared compounds.

The quantum chemical calculations were conducted via the Gaussian trial package Hartree–Fock method to study the electronic structures (E_HOMO_ & E_LUMO_), the energy gap (ΔE), and the molecular geometry of the prepred ILs, as shown in Fig. 4. The quantum calculations were conducted using the basis set 6-31G. As shown in Table 6, increasing the length of the alkyl chain of R_8_-IL to R_14_-IL is directly proportional to the values of E_HOMO_ , E_LUMO_, and softness (σ), while it is inversely proportional to ΔE and dipole moment (µ) values. Figure 4 shows the ability of the prepared ILs to act as electron donors to asphaltene compounds. The dispersion reactivity of the ionic liquid towards asphaltene aggregates is determined by ΔE, where the efficiency of the prepared ILs is increased by decreasing the ΔE of each compound. Therefore, R_14_-IL > R_12_-IL > R_10_-IL > R_8_-IL in reactivity which lowers the needed energy to move electrons from HOMO to LUMO. This is because the smaller the energy gap (ΔE), the easier the absorption between the ionic liquid and the surface of the asphaltenes, which is in turn better for the dispersion efficiency of the ionic liquid. R_10_-IL recorded a lower ionization energy (I) so, it indicates the highest dispersion potential against asphaltene molecules. R_14_-IL possesses the highest dipole moment (µ), while R_8_-IL has the lowest value, as shown in Fig. 5. Dipole moment is releated to the molecule’s global polarity, so the compound with a higher dipole moment value shows more reactivity. Softness is another term that demonstrate the reactivity of the compounds, where the soft compounds indicate more reactivity than the hard molecule’s, therefore R_14_-IL > R_12_-IL > R_10_-IL > R_8_-IL in reactivity .

**Table 6:** Quantum chemical parameters of the synthesized compounds.

Parameter	ILs			
	R_8_-IL	R_10_-IL	R_12_-IL	R_14_-IL
E_HOMO_, eV	−1.296	−1.271	−1.315	−1.316
E_LUMO_, eV	−0.713	−0.729	−0.78	−0.812
Energy gap ΔE, eV	0.583	0.542	0.535	0.504
Dipole moment µ, Debye	7.9	12.6	17.8	23.3
Electron affinity A, eV	0.713	0.729	0.78	0.812
Ionization Energy, eV	1.296	1.271	1.315	1.316
Electronegativity, eV mol^−1^	1.004	1	1.047	1.064
Hardness ɳ, eV mol^−1^	0.291	0.271	0.267	0.252
Softness s, eV^−1^	3.4305	3.69	3.7383	3.9682
Electrophilicity, (ω)	1.73	1.84	2.05	2.24

### Surface tension measurements of the prepared ILs

**Figure 6:**
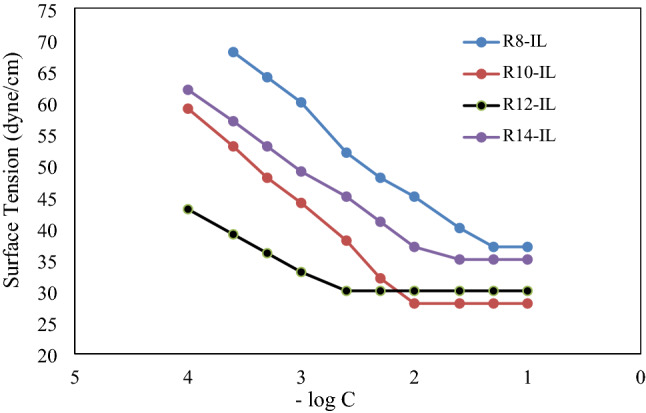
Relation between the surface tension and −log concentration of ILs.

The values of critical micelle concentration (CMC) and the surface tension at CMC of each IL are presented in Table 7, In addition to the interfacial tension (IFT), and the area per molecule at the surface (A_min_). All these data were obtained from Fig. 6, in which the surface tension is ploted against minus the logarithm of the concentrations of the prepared ionic liquids (of R_8_-IL, R_10_-IL, R_12_-IL, and R_14_-IL).

**Table 7:** Surface active parameters of the prepared ILs.

Cpds	CMC mol/L	γ_CMC_ mN/m	IFT mN/m	π_CMC_ mN/m	Pc_20_	Г_max_ × 10^10^mol/cm^2^	A_min_ nm^2^	G^o^_mic_ KJ/mol	G^o^_ads_ KJ/mol
R_8_-IL	4 × 10^–2^	37	7	35	3 × 10^–3^	2.939	0.565	−15.932	−27.842
R_10_-IL	9 × 10^–3^	28	5	44	2.8 × 10^–4^	2.911	0.570	−23.315	−38.429
R_12_-IL	2.3 × 10^–3^	30	4	42	1 × 10^–5^	1.755	0.946	−30.635	−54.564
R_14_-IL	1.5 × 10^–2^	35	4	37	6 × 10^–4^	2.250	0.737	−20.786	−37.210

Surface parameters of the prepared imidazolium-based ionic liquids, such as surface tension (γcmc), interfacial tension (IFT), Critical Micelle Concentration (CMC), surface excess (max), and minimum surface area per molecule (A_min_) were calculated using surface tensiometry and are listed in Table 7.

#### Surface tension (γ) and critical micelle concentration

At 25 °C, Fig. 6 shows the variation of surface tension against -log concentration of the prepred ILs. At the same concentration, the length of the attached alkyl chain of the prepared ILs has a significant effect on the surface active parameters. Where, increasing the length of the alkyl chain to 12 methylene groups reduces the surface tension dramatically. While the IL that has a short alkyl chain (R_8_-IL) displayed a reduced dropping in the surdface tension. This is due to the longer alkyl chain has a greater proclivity to be adsorbed at the air–water interface^42^. Due to the variations in polarity between the hydrophobic alkyl chainand the aqueous medium, the repulsion between the two is amplified by increasing the amount of the repeated methylene groups. At increasing the concentrations of the ILs, the surface tension curve values essentially remain constant, revealing the critical micelle concentration values for each IL. The data in Table 7 revealed that the interfacial tension of the ionic liquids under consideration was decreased with increasing the length of the alkyl chain. The values of critical micelle concentration were clearly reduced when the length of the hydrophobic chain was increased. Because of the long alkyl chains (> 12 methylene groups) have the tendency to be coiled, the CMC value can be easily negatively affected with no additional discernible effects.

#### The efficiency (PC20) and the effectiveness (π_cmc_)

Table 7 shows the efficacy values of the prepared ionic liquids. It is clear that increasing the length of the hydrophobic alkyl chain, increasing the efficiency. This is due to the adsorption efficiency at surfaces is directly proportional to the number of methylene groups in the hydrophobic alkyl chain. The effectiveness (π_cmc_) of the surface tension is determinmed by the surface tension of the IL solution at critical micelle concentration (γcmc). R_12_-IL has the most efficient surface active parameters as it has the lowest γcmc. Adsorption effectivness has a significant role in influencing the characteristics of surfactants, including foaming, wetting, and emulsification^43^.

#### Maximum surface excess (Γ_max_)

The values of Γ_max_ of the prepared ionic liquid is decreased by increasing the number of methylene groups as shown in Table 7. It is known that the activity of surface active agent increases by decreasing the surface tension^44^. One of the most important uses of surface active compounds as a crucial field of chemistry in many applications involves pumping the compound to the interface to generate and adsorbed layer^45^. The data revealed that R_12_-IL has the large area per molecule at the surface, indicating that molecules with long alkyl chains are flexible and less tightly pace at the air–water interface^46^.

#### Thermodynamics of energy adsorption

Data from Table 7 revealed that the prepared ILs have negative values of standard free energy for both micellization and adsorption, indicating that both happened spontaneously. This is due to the presence of repulsion forces between the polar solvent and the hydrophobic alkyl chains. The adsorption process is more preferred than micellization because the adsorption energy is lower than the micellization energy of each ionic liquid.

The prepared ionic liquids showed high negative values of free energy of adsorption, which encourages their utilization in many important applications such as asphaltene dispersion and inhibition, corrosion inhibition, and antimicrobial utility.

The larger values of ΔG^o^_ads_ than those of ΔG^o^_mic_ indicate that the adsorption process at the solution/air interface is more preferable than the micellization process in the bulk of the solution^47,48^.

### Evaluation of the synthesized dispersants

In this work, the synthesized ionic liquids R_8_-IL, R_10_-IL, R_12_-IL, and R_14_-IL were assessed as dispersants for asphaltene aggregates using the viscometric and the refractive index methods. We believe that our research is the first to test asphaltene dispersants utilizing dead oil using the refractive index approach.

#### Viscometric method

**Figure 7:**
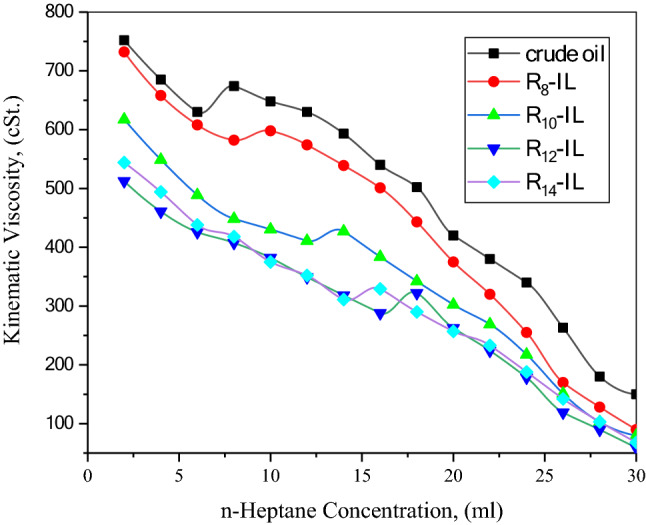
Effect of the prepared dispersants (R_8_-IL, R_10_-IL, R_12_-IL, and R_14_-IL) on the asphaltene onset precipitation.

This technique is predicated on the notion that crude oil containing asphaltene is a colloidal solution in which asphaltenes are the suspended particles and the resin constituent serves as the asphaltene stabilizer. This stabilized system might become upset in the event of the introduction of a precipitant such as n-heptane that has the ability to desorp the resin preserver film from the asphaltene surfaces. Without the presence of resin, the asphaltene molecules have the ability to interact with each other to form aggregates of different sizes. The effect of the asphaltene precipitator (n-heptane) on the onset of precipitation with and without the prepared ionic liquids (R_8_-IL, R_10_-IL, R_12_-IL, and R_14_-IL) is shown in Fig. 7. The kinematic viscosity of the titrant mixture (asphaltenic crude oil and n-heptane) decreased while the concentration of n-heptane increased until it reached a deviation point. After that, a small increase in one point was observed, followed by a great decrease in the viscosity of the titrant mixture. This point of deviation is known as the asphaltene onset precipitation^49^. This point was observed in the blank crude oil after adding 6 ml of n-heptane. This volume was increased to 10, 12, 16, and 18 ml after using 1000 ppm of each R_8_-IL, R_10_-IL, R_12_-IL, and R_14_-IL, respectively. Table 8 contains the needed concentration of n-heptane to detect the onset precipitation point of asphaltene after using different concentrations of the preapared dispersants. Increasing the concentration of the ionic liquids positively affects the onset of asphaltene precipitation. It is obvious that the onset precipitation point of asphaltene was delayed while the length of the attached alkyl chain to the dispersant was increased. However R_14_-IL contains 14 carbon atoms and has the longest alkyl chain, R_12_-IL is more effective as an asphaltene dispersant. This is attributed to the coiling effect of the extra-long alkyl chain in R_14_-IL^50^.

**Table 8:** Effect of different concentrations of the prepared dispersants on the onset precipitation point of asphaltene.

Concentration (ppm)	Onset of precipitation @ (n-Heptane, ml)			
	R_8_-IL	R_10_-IL	R_12_-IL	R_14_-IL
500	8	10	14	12
1000	10	12	18	16
2000	14	16	18	18

#### Refractive index method

**Figure 8:**
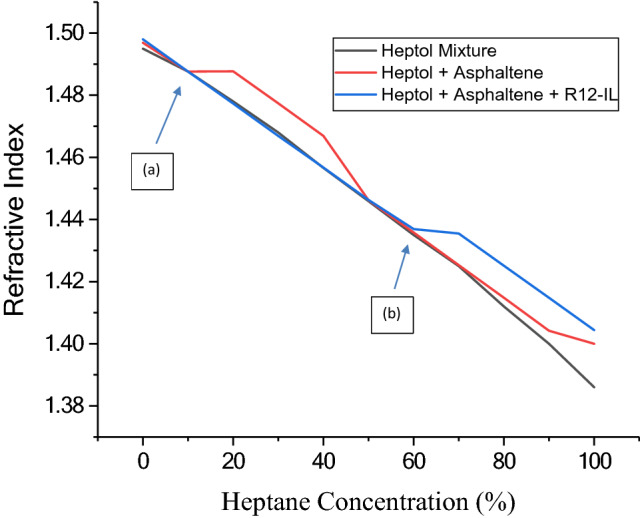
Asphaltene onset precipitation using the refractive index technique, where (**a**) represents the asphaltene onset precipitation in heptol solution, and (**b**) represents the asphaltene onset precipitation in the presence of R_12_-IL.

Precipitation of asphaltenes decreases the polarity properties of the mixture and consequently the value of the refractive index. Increasing the percentage of the precipitant (n-heptane) in the Heptol mixture reduces the refractive index of the mixture in a linear trend as shown in Fig. 8. This behavior also appeared for the asphaltene/heptol mixture until the asphaltene onset precipitation was detected. After reaching this point, the polarity of the remaining asphaltene/heptol mixture decreased, and this may be attributed to the reduced amount of suspended asphaltene molecules. Therfore, the values of refractive index was deviated from its linear trend. The deviation appeared at low concentration of n-heptane as shown in Fig. 8 at point (a) without dispersant, while at point (b) the onset precipitation of asphaltene appeared at nearly 60% of the precipitant after using the ionic liquid as a dispersant.

In order for ionic liquids to stabilize the asphaltene in the media, two key processes must take place: first, the adsorption of the ionic liquid polar head on the surface of asphaltene molecules due to the acid-base interaction between the ionic liquid and the asphaltene molecules, and second, the formation of a stable non-polar alkyl layer around the asphaltene molecules^32^. Consequently, ionic liquid can operate as a bridge between polar asphaltene molecules and the non-polar medium of the crude oil. In general, asphaltene inhibitors are characterized by their high aromaticity and polarity. Therefore, the alkylated ionic liquids seem to be more superior than conventional aromatic inhibitors.

## Conclusion

Four different ionic liquids, named R_8_-IL, R_10_-IL, R_12_-IL, and R_14_-IL, were synthesized and well characterized using different spectroscopic methods such as elemental analysis, FT-IR, and proton NMR. Moreover, the thermal stability of prepared ILs was measured and showed a high level of thermal stability. In addition, the surface tension was measured and showed good surface active poperities. A quantum study of the ionic liquids was conducted to investigate the quantum parameters such as E_HOMO_ and E_LUMO,_ the energy gap, and the geometry optimization of the electronic structure. It was found that the reactivity of the prepared ILs increased with the number of the attached methylene groups.

The prepared ILs were tested to disperse the asphaltene agglomerates using the viscosity and the refractive index techniques. With regard to the all ionic liquids, the strength of dispersion was increased with increasing the concentration of the prepared ionic liquids up to 2000 ppm. This suggests that increasing the amount of the ionic liquid, increases its adsorption on the surface of asphaltene micelles. R_12_-IL showed its high value of inhibition at only 1000 ppm, so the optimum concentration is 1000 ppm.

The dispersion behavior has a regular tendency, with increasing the number of the attached methylene groups increasing the efficiency of dispersion. Because of the coiling effect of the extra-long alkyl chains, this trend loses its relevan as the length of the alkyl chain exceeds 12 carbon atoms.

## Supplementary Information

Supplementary Information.

## Data Availability

The data that support the findings in the present study are available from the corresponding author upon request.
