# Supplementary material for: Investigation the effect of different ionic liquids based-aryl imidazole on the onset precipitation of asphaltene
Source: Sci Rep. 2023 Mar 11;13:4054. doi: 10.1038/s41598-023-31066-0 (PMC10008557; doi:10.1038/s41598-023-31066-0)
Supplement: Supplementary file 1 — Supplementary Information. [file 41598_2023_31066_MOESM1_ESM.pdf]

# Investigation the Effect of Different Ionic Liquids Based-Aryl Imidazole on The Onset Precipitation of Asphaltene”

Raghda A. El-Nagar[1], Maher I. Nessim[1], Dina A. Ismail [2], Manal G. Mohamed[3], Alaa Ghanem[4,*]

[1] Petroleum Testing Lab, Analysis & Evaluation Department, Egyptian Petroleum Research Institute, Nasr City, Cairo, 11727, Egypt.

[2] Surface active agent lab, Petrochemical Department, Egyptian Petroleum Research Institute, Nasr City, Cairo, 11727, Egypt.

[3] Polymer Lab, Petrochemical Department, Egyptian Petroleum Research Institute, Nasr City, Cairo, 11727, Egypt.

[4] PVT lab, Production Department, Egyptian Petroleum Research Institute, Nasr City, Cairo, 11727, Egypt.

Corresponding Email: alaa_ghanem2001@yahoo.com & alaa_ghanem2001@epri.sci.eg

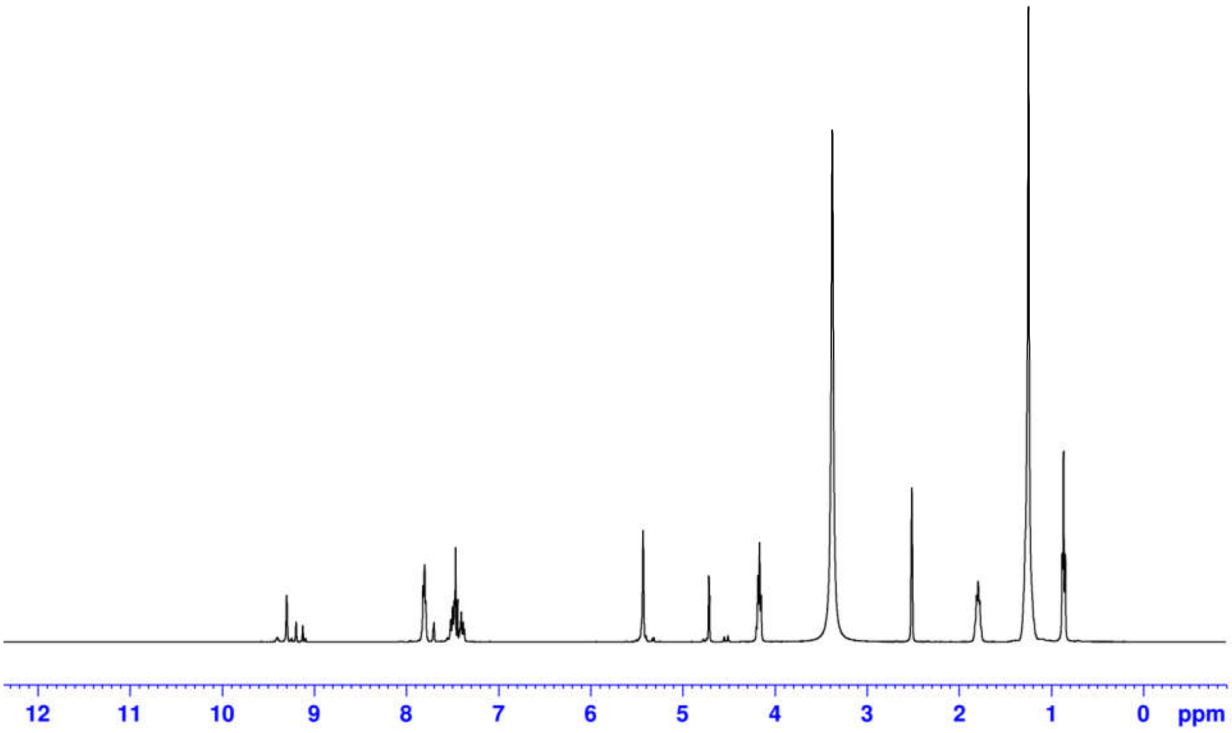


**Figure S1** $^{1}$H NMR spectra of $R_8$-IL

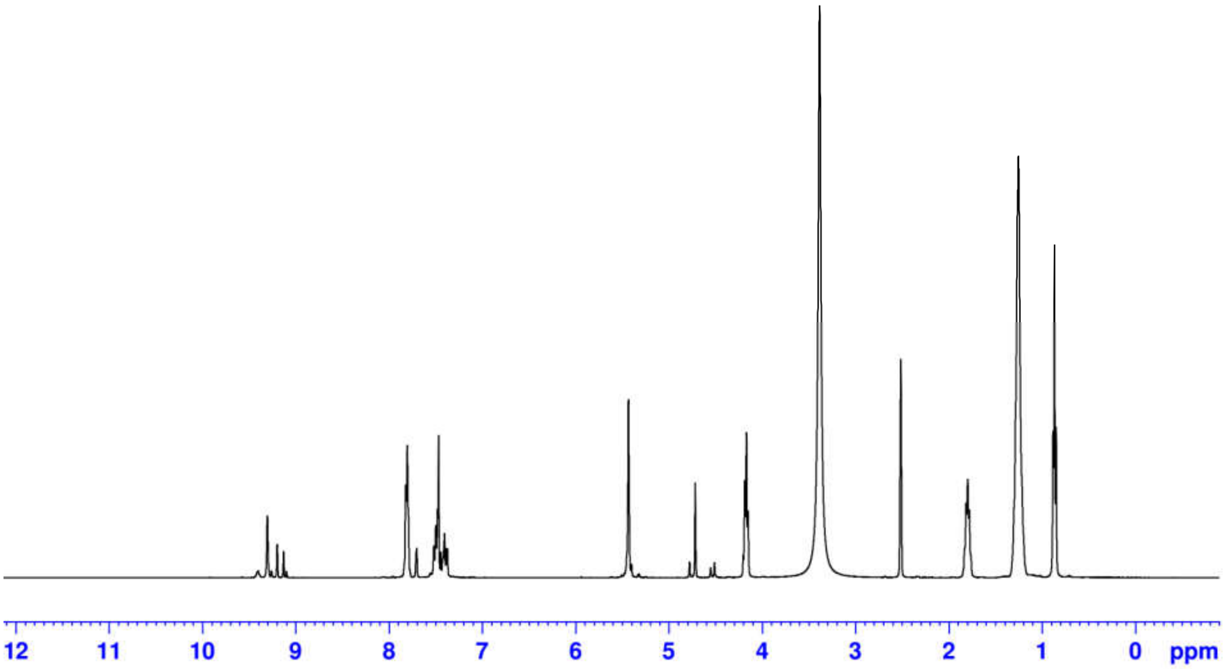


**Figure S2** $^{1}$H NMR spectra of $R_{10}$-IL

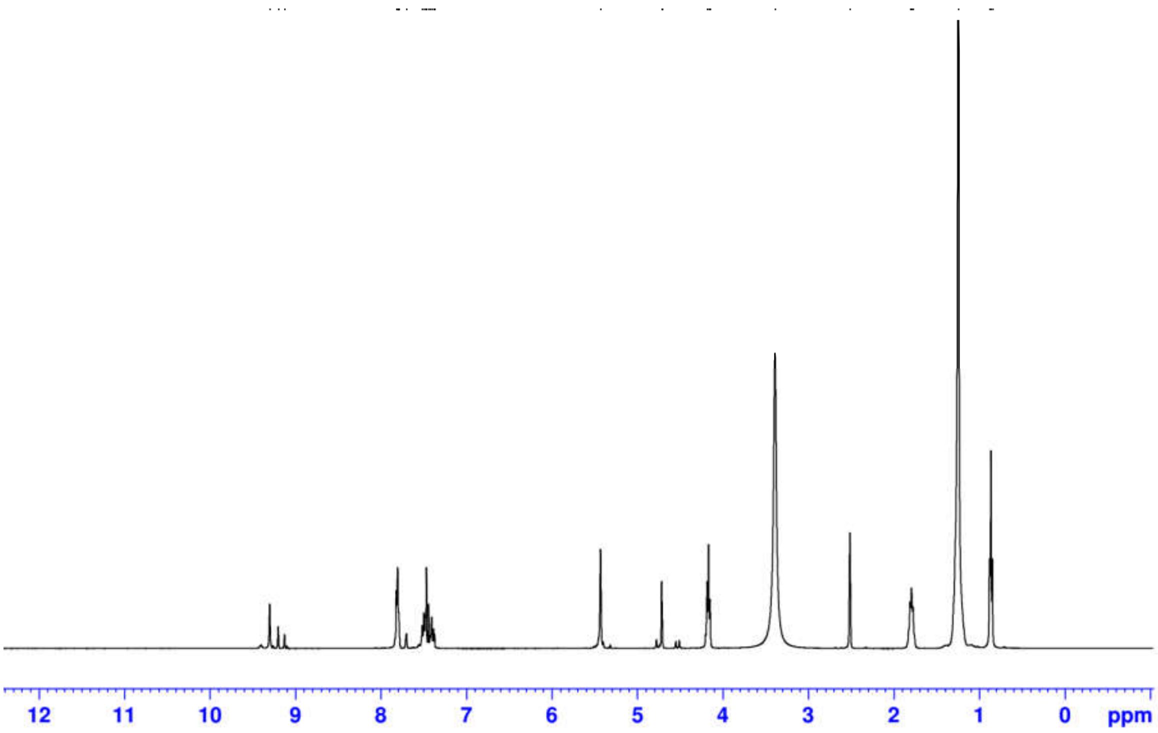


**Figure S3** $^{1}$H NMR spectra of $R_{12}$-IL

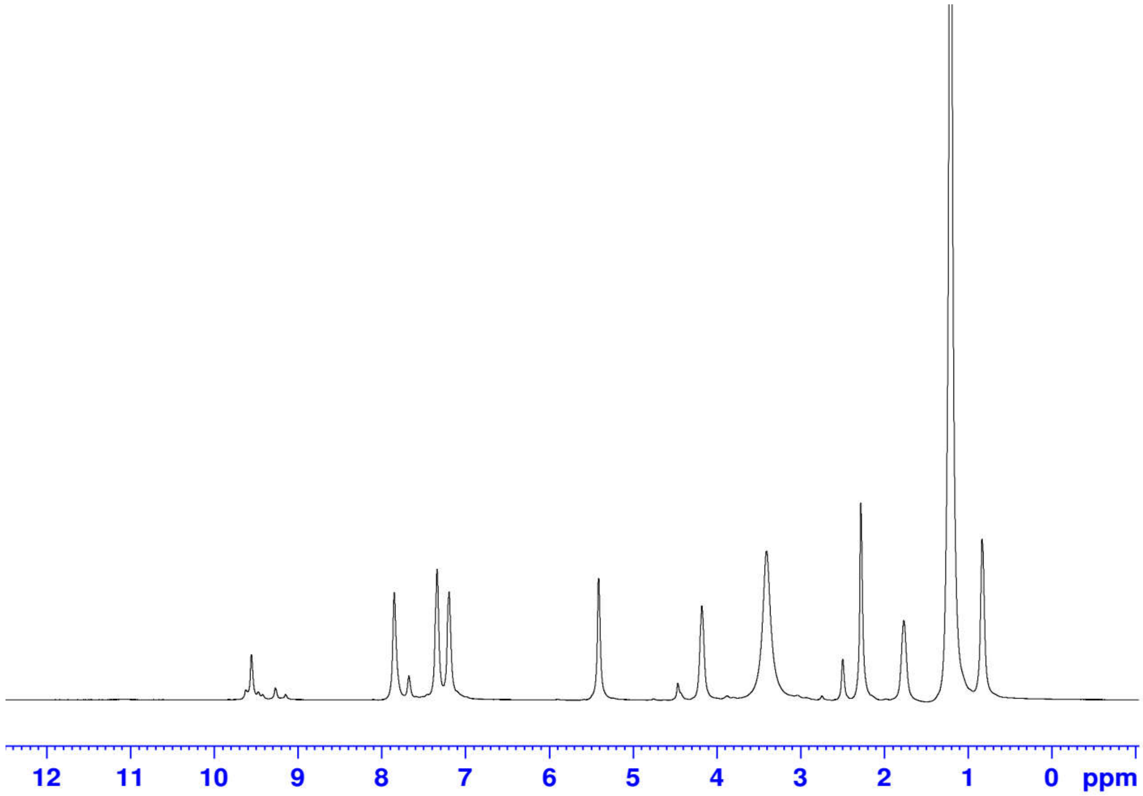


**Figure S4** $^{1}$H NMR spectra of $R_{14}$-IL
